# Enhancing the quality and efficiency of regulatory science literature reviews through innovation and collaboration with library and information science experts

**DOI:** 10.3389/fmed.2024.1434427

**Published:** 2024-07-03

**Authors:** Elizabeth R. Stevens, Gregory Laynor

**Affiliations:** ^1^Department of Population Health, New York University Grossman School of Medicine, New York, NY, United States; ^2^Health Sciences Library, New York University Grossman School of Medicine, New York, NY, United States

**Keywords:** meta-research, literature review methods, information retrieval, regulatory science, collaboration

## Introduction

The importance of literature reviews as part of regulatory science is widely understood. From large trials to follow-up case studies, capturing existing research is key to creating safe and effective regulatory policies. With a shift toward incorporating additional evidence-types—beyond randomized controlled trials (RCTs)—into regulatory decisions and the potential for literature reviews themselves to generate new data (1–7), the importance of high-quality literature reviews is increasing. However, rigorous and comprehensive literature reviews—that ensure important data are not missed (8)—take substantial effort and can be difficult to do well. To encourage both comprehensiveness and efficiency in regulatory science literature reviews, additional focus is needed to integrate rigorous information science methodologies into regulatory science search strategies, as well as to develop innovative tools and strategies that can augment traditional search methods.

Meta-research (research on research) provides a way to examine the efficiency, quality, and potential bias in the overall research ecosystem (9). By applying a meta-research lens to literature reviews in regulatory science, we seek to elaborate on the challenges to performing high quality literature review data retrieval in regulatory science and identify potential strategies that may be explored to improve literature review quality and efficiency. Specifically, we hope to highlight the value of engaging library and information science expertise in the data retrieval process, including the strategic development of new controlled vocabulary and enhancement of conventional search techniques with novel digital technologies.

## Challenges facing literature review in regulatory science

Due to the scope of potentially relevant materials, regulatory expectations, and an increasingly large body of research (10), regulatory science researchers face particular challenges to successfully identifying and retrieving the data they need. Identifying the potential obstacles to effective and high-quality literature review, both unique to regulatory research, as well as those experienced by other disciplines, is important for determining potential solutions to these barriers. The challenges identified fall into two main categories: diversity of potential data sources and breadth of potential search terms (11). These challenges are then overlain by the various stipulations put forth by regulatory bodies, such as post-marketing safety reports (12), thus, contributing to the importance of rapid turn around and the need for repeated searches (13).

Data relevant to a specific regulatory science research question may be found throughout a multitude of databases and repositories that house peer-reviewed research studies and/or gray literature (14). Some databases have a broad topical and geographic focus, whereas others may be more specialized or include language-specific resources. While there is often significant overlap among database contents, some materials will only be found in select databases; this necessitates a detailed knowledge of database characteristics to identify relevant search locations, as well as a search strategy that spans multiple databases (15).

Gray literature can require an additional level of awareness and expertise to identify, as these non-peer-reviewed data sources are often not indexed in the same manner as peer-reviewed literature. Conference proceedings, institutional reports, interim results, and other types of gray literature may be the first data sources to publish important adverse reaction outcomes and therefore may be necessary to identify. These materials, however, are found in a diverse set of platforms that are not always well-known or easily searchable (16). Similarly, relevant data may be found as secondary analyses within published clinical studies. Thus, additional expertise may be required to search beyond a title or abstract to identify potentially relevant literature. Furthermore, not all published materials are of equivalent quality, and in the age of paper mills, predatory journals (17), fraudulent citations (18), and paper retractions (19), having the expertise to discern which sources are reliable is needed to produce a high quality regulatory literature review (20, 21).

In addition to the diverse locations in which it can be found, identifying relevant regulatory data can require an extensive list of search terms, as there can be inconsistency across geography, disciplines, and historically in how similar topics are described. To capture literature relevant for answering a research question, identifying an appropriate set of search terms is a critical step (22, 23). Additionally, databases often employ a controlled vocabulary for indexing articles (such as PubMed's Medical Subject Headings, MeSH). A search strategy encompassing multiple databases will often require translating the search into the controlled vocabulary of each database (24). Defining search terms, can also pose a particular challenge depending on whether a topic has differing terminology in multiple disciplines, terminology that has changed over time, or a limited maturity of the research field leading to less well-characterized key terms (25, 26).

Regulatory bodies often have requirements for the collection of data published in other countries, all drug or device formulations, and across the development spectrum. This can contribute to the need to identify appropriate vocabulary that accounts for foreign languages, spans all stages of development from pre-clinical to clinical studies, includes synonyms and older terms that have been replaced by more current terms, and captures all ways in which related products may be labeled. Furthermore, as the field of regulatory science has sought to include non-RCT evidence (1–7), additional vocabulary is needed to identify alternate study designs and data sources.

## A role for library and information science experts

While in the age of rapid online literature searching the role of a librarian or other information science experts in literature reviews may feel unnecessary, the role of these experts has become even more vital to the production of high quality, thorough, and expedient regulatory science literature reviews. Finding all of the existing relevant research is a key component in performing effective regulatory research. However, with a multitude of databases and variations in search terms, understanding how and where to identify relevant data is not a simple task and requires strategy and knowledge of data systems. Therefore, studies that choose to include those with library and information science expertise on their teams are at a significant advantage for producing high quality literature reviews with less overall effort (27–29).

Indeed, a whole field of library and information science has evolved to develop methods for the management and discoverability of research data for research synthesis, whether in the published literature or in data repositories (30). Those skilled in information science can provide invaluable insights into the best approaches to identify potentially relevant literature (27–29). Information science experts can improve how controlled vocabulary can be used or improved for a specific research topic, as well as generate high quality search strategies.

Potential solutions to reduce the burden of information retrieval and maximize the quality of regulatory science literature reviews may be found in the expanded use of existing controlled vocabulary and strategic development of new controlled vocabulary. The use of controlled vocabulary, or indexing terms, can facilitate and improve the accuracy of literature searches being performed (31, 32), and are a powerful tool that can overcome some of the limitations seen in a basic keyword search. Without the use of controlled vocabulary, search results would be limited to specific keywords or phrases used in the text, which could lead to irrelevant or incomplete results due to inconsistences in terminology used or alternate spellings. Thus, the standardization provided by controlled vocabulary helps ensure research is discoverable and accessible to a wider audience. Therefore, having knowledge of the controlled vocabulary for each database is a valuable skillset that can improve the comprehensiveness of a literature review. However, controlled vocabulary does have its limitations and not all important areas of research have an existing controlled vocabulary available (33, 34). Furthermore, gray literature sources are typically not indexed with controlled vocabulary. Librarians or those with an information science expertise can assist in search strategies to overcome the shortcomings of controlled vocabulary, as well as help advance the strategic development of new controlled vocabulary specific to regulatory research (35, 36).

## A role for technological innovation

Even with a meticulously designed search strategy, the sheer amount of research findings in existence creates an important challenge to successfully and efficiently identifying all relevant research. Technological innovations, such as natural language processing (NLP) and generative artificial intelligence (GenAI), have the potential to provide alternative or supplementary approaches for identifying relevant research studies to address gaps and inefficiencies in information retrieval. Automation may help address the high volume of items in need of screening due to search term limitations by increasing search specificity and reducing the workload burden associated with manually screening all potentially appropriate items identified during literature search (37). However, there is significant work to be done before automation tools are viewed as successfully replacing traditional search methods (38, 39).

During the literature search process, a balance must be struck between achieving the sensitivity necessary to identify all relevant documents while maintaining specificity to reduce the amount of irrelevant data collected. When using classic systematic search strategies aimed to maximize sensitivity, this can result in a large number of irrelevant documents that must be screened manually. Despite the importance of comprehensive information retrieval for high quality literature reviews, much of the effort placed into automating regulatory science data retrieval has been focused on enhancing the efficiency of article screening and data extraction (40), rather than improving the comprehensiveness and quality of data being collected in the literature search itself. Efforts for automating the review need to not just decrease workload but also improve the quality of the data being identified (41).

For topics and data sources that do not have an extensive controlled vocabulary, NLP tools can potentially assist researchers by providing an alternate method for identifying relevant literature. NLP combines computational linguistics, machine learning, and deep learning models to process human language to allow a computer program to understand human language as it is spoken and written, rather than relying on predefined vocabulary or the presence of specific phrases or terms within the text. This type of automation can equip a researcher to increase the specificity of their search strategy while maintaining sensitivity, as NLP can capture a broader range of vocabulary combinations, while providing phrase interpretation to eliminate data sources that use similar vocabulary, but in irrelevant contexts.

During the regulatory research literature review process, additional sources of information may be gleamed from within the reference lists of identified documents (42). But identifying and evaluating these citations can be time consuming and existing technology approaches have demonstrated suboptimal performance (38, 43, 44). GenAI tools can be developed to scan through research documents to identify and extract potentially relevant citations from a document's reference section for further review (43). Furthermore, the summarization capabilities of GenAI can reduce the burden of the screening process. Relevant data in a document may be contained within secondary analyses or sub-studies that are not reported in the document title or abstract, making the identification of these data sources time consuming, as they require a full-text review. GenAI tools can be developed to analyze the full text and provide a summary of the data to minimize the effort required to screen these sources. While some screening and summarization tools are available, many have not been validated for the literature review data extraction and screening process (39, 45–49). Collaboration with an information science expert may improve the process of developing tools with improved validity.

To truly make these technologies work for regulatory science research, however, concerted investment is needed to develop and adapt these tools to meet the needs of the regulatory science researcher and meet systematic review standards (39). A collaboration between regulatory scientists and information science experts can serve to facilitate the development of effective and relevant review tools. To ensure reliability, GenAI and NLP approaches need to be validated on search use cases. However, the creation of datasets for training and validation can be time intensive and requires existing literature search expertise (50). In additional to collaborating on tool development, librarians and data science experts can be an important resource to evaluate the validity and utility of tools being developed within industry. Furthermore, attention must be given to the current limitations of using even “well-trained” GenAI. While adept at information generation and summarization, GenAI cannot discern the quality of the data sources it pulls from (51). Therefore, additional training will likely be necessary for a GenAI model to distinguish high- and low-quality data sources from an information science perspective (e.g., predatory journals) as well as discern what is a high-quality data source as per standards set forth by regulatory bodies.

## Conclusion

Finding all existing relevant research is a key component to performing effective regulatory science research. However, regulatory science researchers face particular challenges to identifying and retrieving relevant data in an increasingly large body of research findings. With a high burden associated with sifting through irrelevant sources and a growing number of resources that may not be identified when using traditional or less sophisticated search strategies, performing high quality and efficient regulatory science reviews is a challenge. To address these barriers, further efforts are needed to increase the integration of rigorous information science expertise into regulatory science search strategies, as well as to develop innovative tools and strategies that can augment traditional search methods.

## Author contributions

ES: Conceptualization, Methodology, Writing – original draft. GL: Conceptualization, Methodology, Writing – review & editing.
